# Investigating the Effects of Paralinguistic Configuration in Background Human Speech on Attention: Protocol for a Within-Subject Study

**DOI:** 10.2196/93525

**Published:** 2026-09-30

**Authors:** Zilu Liang, Carl Daniel Karlsson

**Affiliations:** 1 Ubiquitous and Personal Computing Lab, Kyoto University of Advanced Science, 18 Yamanouchi Gotanda-cho, Ukyo-ku, Kyoto, 615-8577, Japan, 81 754966510; 2 Institute of Industrial Science (IIS), The University of Tokyo, Tokyo, Japan; 3 Technical University of Denmark, Kongens Lyngby, Denmark

**Keywords:** background noise, attention, multimodal sensing, paralinguistics, cognitive performance, psychophysiology, pupillometry, electrodermal activity, auditory distraction

## Abstract

**Background:**

Background noise is common in everyday environments; yet, its effects on attention vary according to task demands, individual characteristics, and properties of the noise itself. Previous research has largely focused on music, nonspeech noise, or intelligible speech, leaving the effects of paralinguistic components of human speech (eg, tone, pitch, and rhythm) difficult to disentangle from linguistic content.

**Objective:**

This study aims to investigate whether paralinguistic characteristics of semantically inaccessible background speech influence attentional performance. Specifically, it will compare focused and diffused vocal stimuli that differ in paralinguistic configuration, with silence included as a reference condition, while also examining physiological responses and individual differences.

**Methods:**

Adults aged 18 years or older (target n=60) will complete one 120-minute laboratory session. In a counterbalanced within-subject design, participants will perform attention-related tasks under 3 auditory conditions: silence, focused vocal stimuli, and diffused vocal stimuli. The primary outcome will be the concentration performance (CP) score of the d2 Test of Attention, and reading comprehension accuracy will be a secondary behavioral outcome. Pupillometry, electrodermal activity (EDA), and blood volume pulse (BVP) will be recorded continuously, with mean pupil diameter, phasic EDA response rate, and the root-mean-square of successive differences (RMSSD) designated as prespecified secondary physiological outcomes. Individual differences in working memory capacity, noise sensitivity, attention-deficit hyperactivity disorder (ADHD) symptomatology, sleep quality, and mood will be assessed as potential moderators. The primary confirmatory analysis will use a linear mixed-effects model with auditory condition as a fixed effect, block position as a covariate, and participant as a random intercept. The prespecified primary contrast will compare the focused and diffused vocal conditions at α=.05. Focused versus silence and diffused versus silence will be examined as secondary contrasts with Holm adjustment for multiple comparisons. The Holm procedure will be used to adjust for multiple testing across the prespecified physiological outcomes, while moderation analyses will be considered exploratory. The protocol was refined following a formative pilot with 9 participants using an abbreviated 2-block procedure.

**Results:**

Ethics approval was granted in June 2026. No participants have been recruited for the full study described in this protocol. Recruitment is scheduled to begin in September 2027, with data collection projected to conclude in September 2029, and data analysis expected to be completed by September 2030. Dissemination of the results is anticipated by September 2031.

**Conclusions:**

This protocol provides a reproducible approach for investigating whether paralinguistic characteristics of semantically inaccessible background speech influence attention and for examining accompanying physiological responses. The study will test whether attentional performance differs between focused and diffused vocal conditions and explore individual characteristics that may moderate these responses. The findings may inform future research on auditory distraction and the design of occupational and educational environments.

## Introduction

### Background

Ambient noise is a ubiquitous feature of modern environments, frequently present in classrooms, offices, and homes. Its influence on cognitive performance, particularly attention, has been studied for several decades. Accumulated evidence suggests that the effects of background sound depend jointly on the demands of the concurrent task, the acoustic characteristics of the sound, and the characteristics of the listener. Tasks that place high demands on working memory or sustained attention, such as proofreading or mental arithmetic, are especially susceptible to interference from sounds carrying semantic information or dynamic pitch variations [1]. In contrast, more rule-based or highly structured tasks, such as grammatical judgments or missing-item detection, tend to be comparatively robust to auditory distraction [2]. Responses to background noise also vary substantially across individuals. While some people report improved focus or learning efficiency in the presence of background music or ambient noise [3,4], others experience increased distraction and performance decrements under similar conditions [1]. This heterogeneity has been attributed to multiple interacting factors, including developmental stage (eg, children vs adults), individual cognitive and affective traits, task characteristics, and the acoustic properties of the noise itself [1,2,5-9]. These findings underscore the importance of considering both acoustic and individual factors when investigating the effects of background sound on attention.

This study builds on 3 areas of prior research, each of which leaves an important question unresolved. First, a substantial body of controlled research has examined the cognitive effects of music and stationary nonspeech noise, particularly white and pink noise, and systematic reviews have characterized this literature in considerable detail [1,10]. Research has also examined ambient noise derived from human voices, which more closely resembles everyday environments such as cafés, libraries, and shared workspaces [2,3,7,9]. Meta-analytic evidence further indicates that task-irrelevant speech can interfere with reading performance [11]. However, studies using speech stimuli have generally focused on speech intelligibility, semantic content, or the presence vs absence of speech rather than isolating paralinguistic configuration itself. Consequently, prosodic, rhythmic, and other dynamic vocal features often remain confounded with linguistic meaning, making it difficult to determine whether nonsemantic properties of human voices independently influence attention. Although recent studies have begun to examine paralinguistic speech features in relation to outcomes such as stress recovery [12], their specific role in attentional performance remains poorly understood.

Second, existing theoretical accounts provide different explanations for how background sounds may influence attention. The Moderate Brain Arousal (MBA) model proposes that moderate levels of noise can facilitate cognitive performance by introducing neural variability that may enhance weak signals through stochastic resonance [13]. Under this account, the effects of noise depend partly on baseline arousal and individual characteristics. Individuals with lower baseline dopamine activity, including some individuals with attention-deficit hyperactivity disorder (ADHD), have been hypothesized to benefit more from moderate noise, whereas those with higher baseline arousal may be more susceptible to distraction [4]. The MBA model is therefore concerned primarily with noise-induced arousal and does not, in its standard formulation, distinguish effects arising from speech structure from those arising from other sound sources.

Interference-based accounts instead emphasize the structural and attentional properties of sound. Changing-state accounts propose that acoustically varying sequences can interfere with task-relevant serial processing, while attentional-capture accounts emphasize disruption caused by salient or deviant auditory events; the duplex-mechanism account distinguishes these 2 routes to auditory distraction [2,6,8]. Paralinguistically rich but semantically inaccessible speech provides a useful case for examining these different explanations because it retains dynamic acoustic properties while minimizing accessible linguistic meaning. From an interference perspective, paralinguistic variation may contribute to disruption by creating changing-state or attention-capturing signals, whereas the MBA model emphasizes noise-induced arousal and individual differences rather than speech structure per se. Examining nonsemantic vocal stimuli may therefore help clarify the conditions under which these mechanisms contribute to attentional performance.

Third, limitations in existing measurement approaches make it difficult to determine the processes underlying observed behavioral effects. Accuracy and response time can establish whether performance differs across auditory conditions but provide limited information about accompanying changes in cognitive effort or physiological arousal. Psychophysiological measures can provide complementary information about these responses. For example, pupil dynamics are associated with arousal and cognitive effort, electrodermal activity (EDA) with sympathetic activation, heart rate variability with autonomic activity, and spontaneous blink rate with aspects of attentional state [14-16]. Studies combining behavioral and physiological measures have demonstrated the value of such multimodal approaches in occupational and applied settings [15,16], but they remain uncommon in the background-speech literature, where physiological investigations have more often focused on affective or stress-related outcomes than on sustained attention [12]. In addition, individual characteristics including noise sensitivity, working memory capacity, sleep quality, and mood have been associated with subjective or cognitive responses to background noise [17-19]; yet, they have rarely been examined together within a common experimental framework.

These gaps motivate our experimental design, which isolates differences in paralinguistic configuration while minimizing semantic accessibility and examines behavioral and physiological responses under controlled auditory conditions. Our protocol also measures individual differences that may contribute to variability in responses to background speech, allowing their potential moderating roles to be explored.

### Formative Pilot Work Informing the Present Protocol

Before finalizing the present protocol, we conducted a formative pilot with 9 participants using an abbreviated procedure comprising the N-back task and 2 of the 3 auditory-condition blocks. The pilot used a separate sample and was conducted solely to assess procedural and technical feasibility, calibrate the newly developed reading comprehension task, determine whether the auditory stimuli were perceptually distinguishable, and refine procedural parameters. It was neither designed nor powered to test the study hypotheses, no inferential analyses were performed, and the pilot data will not contribute to the full study described in this protocol.

The pilot demonstrated that the behavioral tasks and concurrent eye-tracking and wrist-worn physiological recording could be completed within a single laboratory session. Participant feedback and task responses were used to refine the reading comprehension materials and indicated that the focused and diffused auditory stimuli were perceived as qualitatively distinct. Observations regarding playback-volume selection and task recovery also informed retention of the available volume range and adoption of a 5-minute rest interval between experimental blocks.

### Objectives

The primary objective of this study is to investigate whether paralinguistic characteristics of background human speech influence attentional performance. Specifically, the study will compare focused and diffused vocal stimuli that differ in paralinguistic configuration while minimizing semantic accessibility, with silence included as a reference condition.

The primary analysis will examine whether attentional performance, measured by the d2 concentration performance (CP) score, differs between the focused and diffused vocal conditions. Reading comprehension will be examined as a secondary behavioral outcome. Secondary analyses will examine physiological responses across auditory conditions using pupillometry, EDA, and heart rate variability. The study will also explore whether individual differences, including noise sensitivity, ADHD symptomatology, working memory capacity, sleep quality, and mood, moderate behavioral and physiological responses to the vocal conditions.

The study is guided by the following hypotheses:

- H1: Effect of paralinguistic characteristics on attention Attentional performance, as measured by the d2 CP score, will be lower under the focused vocal condition than under the diffused vocal condition.
- H2: Physiological correlates of auditory condition The focused and diffused vocal conditions will be associated with differences in physiological state and task-related physiological responses, as assessed using mean pupil diameter, phasic EDA response rate, and the root-mean-square of successive differences (RMSSD).
- H3: Moderating role of individual differences Individual differences in noise sensitivity, ADHD symptomatology, working memory capacity, sleep quality, and mood may moderate the effects of vocal condition on attentional performance and physiological responses.

## Methods

### Study Design

This study is a nonrandomized, within-subject laboratory experiment involving 3 counterbalanced auditory conditions: focused vocal stimuli, diffused vocal stimuli, and silence. Applicable items from the SPIRIT (Standard Protocol Items: Recommendations for Interventional Trials) 2025 reporting guideline were used to support comprehensive reporting of the protocol (Checklist 1) [20]. Figure 1 illustrates the overall experimental procedure and timeline.

**Figure 1.**
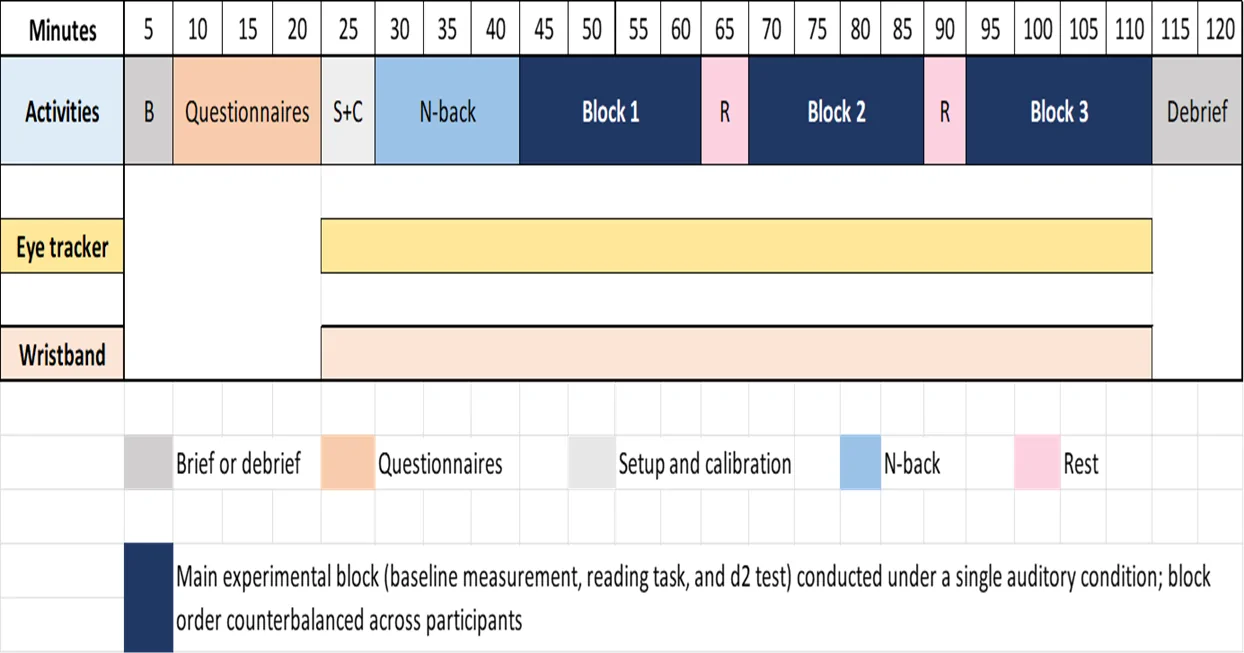
Experimental procedure and timeframe.

### Patient and Public Involvement

Members of the public were not formally involved in the design, conduct, or reporting of this study. Participant experience nonetheless informed the final design. Feedback from the 9 formative pilot participants on session length, task difficulty, stimulus volume, and the perceived character of the auditory conditions directly shaped the session structure, the retained 20‐65 dB volume range, and the 5-minute rest intervals, as described in the Introduction. Public involvement in interpreting and disseminating the findings will not be planned beyond the participant summary described under dissemination policy.

### Ethical Considerations

Ethics approval for the study was obtained from the Ethics Review Board of Kyoto University of Advanced Science (approval number 26E01; June 22, 2026). The separate formative pilot described in the Introduction was conducted under approval number 22E01. Participation will be entirely voluntary. Prospective participants will receive a written information sheet describing the study purpose and procedures, physiological measurements, expected session duration, data handling, compensation, and their right to withdraw. Written informed consent will be obtained from all participants before participation in the study. Participants will be informed that they may withdraw at any time without providing a reason and without penalty. They will also be informed of the procedures governing data collected before withdrawal, including whether and under what circumstances previously collected identifiable data can be deleted.

Each participant will be assigned a study identifier at enrollment. Questionnaire, behavioral, and physiological data will be stored using this identifier, while the linkage file connecting study identifiers with personally identifying information will be stored separately and accessible only to the principal investigator and authorized study personnel. Analytic datasets will be deidentified, although they will not be considered fully anonymized while the linkage key is retained. Data-management computers will be maintained offline with access controls, and installation of unauthorized software will be restricted. Study personnel with data access will complete institutional information-security training. Raw eye-tracking recordings, including scene-camera footage, and raw physiological recordings will be treated as potentially identifiable or sensitive data and subject to additional safeguards. These data will be stored in encrypted offline media, will not be publicly shared, and will be deleted 5 years after publication in accordance with the institutional retention policy.

Participants will receive a ¥3000 Amazon (ACI Gift Cards, Inc) gift card (approximately US $20) for completing the study session. Participants who withdraw before completing the session will not receive compensation.

### Participants and Eligibility Criteria

Participants will be recruited through convenience sampling, primarily through campus advertisements.

Participants must meet the following criteria: (a) be 18 years of age or older; (b) self-report English proficiency of intermediate level or higher (≥3 on a 5-point Likert scale); (c) be able to attend the in-person experimental session; and (d) be able to complete eye-tracking tasks without wearing glasses (contact lenses permitted).

Participants will be excluded if they self-report (a) a diagnosed neurological disorder, or (b) severe medical or psychiatric disorders that may interfere with task performance or physiological measurements.

No additional restrictions based on demographic characteristics will be imposed. To account for individual differences that could influence cognitive outcomes, participants will complete a battery of psychometric instruments assessing sleep quality, mood, chronotype, ADHD symptoms, dyslexia risk, and noise sensitivity, as well as the N-back test. These measures will be used to characterize individual differences that may influence cognitive performance, with prespecified measures examined as exploratory moderators as described in the analysis plan.

### Sample Size

The sample size was determined for the primary confirmatory outcome, the CP score of the d2 Test of Attention, and the prespecified focused-vs-diffused contrast. This contrast directly addresses the primary hypothesis (H1) that attentional performance differs according to paralinguistic configuration when both conditions contain speech-derived background sound.

No previous study has examined the focused-vs-diffused paralinguistic manipulation used in the present protocol with d2 CP as the outcome, and a directly applicable effect-size estimate is therefore unavailable. The sample-size calculation was therefore based on the minimum standardized within-participant effect that the study should be able to detect. For the prespecified focused-vs-diffused contrast, sample size was calculated for a two-sided paired comparison with α=.05, power of 0.80, and a standardized within-participant effect of dz=0.40. Using the noncentral t distribution, the minimum required sample was 52 participants with complete primary-outcome data. To provide a modest allowance for incomplete or unusable primary behavioral data, we plan to recruit 60 participants. The study may have limited power to detect effects smaller than dz=0.40; effect estimates and 95% CI will therefore be reported alongside hypothesis tests.

The sample-size calculation applies specifically to the primary focused-vs-diffused comparison under H1. The physiological outcomes under H2 were not independently used to determine the sample size because directly applicable effect-size estimates are unavailable for the present stimulus manipulation. The moderation analyses under H3 involve interaction effects and were also not used to determine the target sample size. These analyses are therefore designated exploratory and will be interpreted primarily using interaction estimates and 95% CIs.

### Experimental Procedure and Timeframe

This study adopts a within-subject, single-session experimental design. Each participant will complete 1 laboratory session lasting approximately 120 minutes, during which attention-related tasks are performed under 3 auditory conditions: (1) background noise with focused vocal stimuli, (2) background noise with diffused vocal stimuli, and (3) silence (control). All participants will be exposed to all 3 conditions within the same session. The order of auditory conditions will be counterbalanced across participants using a predetermined schedule to control for order effects. Figure 1 illustrates the experimental timeline, which consists of the following phases.

#### Preexperimental Phase

The session will start with a briefing, followed by a set of questionnaires assessing individual characteristics, including noise sensitivity, attention-related traits, mood, and sleep quality and timing. Participants will then undergo sensor setup and calibration, which includes fitting and calibration of the eye tracker and wrist-worn physiological sensors. A short silent baseline recording will be collected during this phase to verify signal quality and establish physiological reference levels.

#### Baseline Cognitive Assessment

Participants will next complete a single N-back task, which is used to index baseline working memory updating and executive control. This task will be administered once at the beginning of the session and will be treated as an individual-differences moderator rather than as a primary outcome measure during data analysis.

#### Auditory-Condition Task Blocks

Participants will subsequently complete 3 main experimental blocks (Block 1‐3), each conducted under a different auditory condition (silence, focused vocal noise, or diffused vocal noise). To minimize order effects, the sequence of auditory conditions across the 3 experimental blocks will be counterbalanced. With 3 conditions, this yields 6 possible condition orderings, and it will be ensured that each condition appears equally often in the first, second, and third block positions across participants. Participants are assigned to these sequences in a sequential manner upon enrollment to achieve approximately equal representation of each ordering in the final sample.

Each block will consist of a baseline measurement period under the assigned auditory condition, followed by the reading comprehension task and the d2 Test of Attention. At the end of each block, participants will complete subjective ratings of the auditory condition, including the Emotional Salience of Sounds Questionnaire B (ESSQ-B) items and ratings of perceived distraction and fatigue. A 5-minute rest and washout period will be inserted between consecutive blocks to reduce carryover effects related to arousal, fatigue, and physiological activity before exposure to the next auditory condition. The duration of the rest periods was determined based on pilot data and established literature on cognitive recovery [21]. As illustrated in Figure 1, eye-tracking (pupillometry) and wristband-based physiological signals, including EDA and blood volume pulse (BVP), will be recorded continuously from the calibration phase through the end of the third experimental block.

#### Postsession Phase

After completion of the final experimental block, participants will provide open-ended feedback in a debriefing session.

### Measures and Materials

#### Psychometric Instruments

This protocol incorporates several psychometric instruments to characterize individual differences that may be relevant to attentional performance. Table 1 summarizes the psychometric instruments used in the study and their purposes.

Measures of noise sensitivity, ADHD symptomatology, sleep quality, and mood are included as potential moderators of responses to the auditory conditions, and will be examined in exploratory analyses. Working memory capacity, assessed separately using the N-back task, will also be examined as an exploratory moderator. Chronotype and reading history are assessed to provide additional context for interpreting individual differences in task performance.

Specifically, participants will complete the Weinstein Noise Sensitivity Scale (WNSS-21), Adult ADHD Self-Report Scale (ASRS-v1.1), Pittsburgh Sleep Quality Index (PSQI), Brief Mood Introspection Scale (BMIS), Ultra-Short Munich ChronoType Questionnaire (µMCTQ), and Abbreviated Adult Reading History Questionnaire (ARHQ-Brief). The ARHQ-Brief is included to characterize history of reading difficulties that may be relevant to performance on the reading comprehension task. Demographic information will also be collected to characterize the study sample.

A limitation of the psychometric assessment is its reliance on self-report, which may be affected by recall and response biases [22,23]. However, self-report is appropriate for assessing subjective constructs such as noise sensitivity, perceived sleep quality, mood, and ADHD-related symptoms, and the selected instruments have established psychometric properties for their intended research uses. These measures are used to characterize individual differences and support exploratory analyses rather than to provide clinical diagnoses or objective assessments of the underlying constructs. Their relatively low participant burden also makes them suitable for inclusion in the present multimodal protocol.

**Table 1.** Psychometric instruments used in the study.

Instrument	Purpose
Demographic Questionnaire (6 items)	Collects demographic information (age, education, and occupation) to characterize the sample and support exploratory analysis where appropriate.
µMCTQ^a^, 6 items [24]	Assesses chronotype to characterize individual differences that may be relevant to task performance.
PSQI^b^, 19 items [25]	Assesses subjective sleep quality over the previous month as a potential moderator of behavioral and physiological responses.
WNSS-21^c^, 21 items [26]	Assesses individual differences in noise sensitivity as a potential moderator of responses to the auditory conditions.
ASRS-v1^d^.1, 18 items [27]	Assesses ADHD^e^ symptomatology as a potential moderator of responses to the auditory conditions.
ARHQ-Brief^f^, 6 items [28]	Assesses history of reading difficulties that may affect reading comprehension performance.
BMIS^g^, 16 items [29]	Assesses current mood before the experiment as a potential moderator of behavioral and physiological responses.
ESSQ-B^h^, 7 items [30]	Assesses subjective response to auditory stimuli as a manipulation check.

a µMCTQ: Ultra-Short Version of the Munich ChronoType Questionnaire.

b PSQI: Pittsburgh Sleep Quality Index.

c WNSS-21: Weinstein Noise Sensitivity Scale.

d ASRS-v1.1: Adult ADHD Self-Report Scale.

e ADHD: attention-deficit hyperactivity disorder.

f ARHQ-Brief: Abbreviated Adult Reading History Questionnaire.

g BMIS: Brief Mood Introspection Scale.

h ESSQ-B: Emotional Salience of Sounds Questionnaire B.

#### Baseline Cognitive Measure: The N-Back Task

The N-back task [31,32] will be administered solely as a baseline measure of working memory capacity and will be used as a moderator in subsequent analyses. Participants will be presented with a continuous sequence of stimuli and must indicate whether the current stimulus matches the one presented N steps earlier in the sequence. The task will be administered using the open-source Brain Workshop software (Paul Hoskinson and Jonathan Toomim) [33], selected for its accessibility and flexible configuration options. During a tutorial phase, participants will complete 1-back and 2-back trials in both spatial and auditory modalities to familiarize themselves with the task. During the test phase, participants will complete 2-back and 3-back trials in the spatial modality, as well as 1-back to 3-back trials in a dual-modality condition combining spatial and auditory input. Each condition will consist of 29 trials, with each trial lasting 3 seconds.

Accuracy will be recorded for each trial, and summary scores will be calculated for each condition. Although Brain Workshop does not record response times, it was selected over other platforms such as PsyToolkit (Prof. Gijsbert Stoet) due to its user-friendly interface and modifiability.

#### Reading Comprehension Task

An original reading comprehension task was developed specifically for this experiment to measure the effects of background audio on sustained attention and learning. Unlike many online reading comprehension tools, this task was designed to separate encoding and retrieval phases, allowing for a clearer interpretation of auditory effects on learning. Participants will read a long expository passage (~1200 words) under an auditory condition. After a short distraction interval (1 minute), participants will complete a set of multiple-choice questions assessing comprehension and recall. To minimize practice effects, 3 parallel versions of the task with equivalent difficulty will be used across auditory conditions. The reading comprehension task was piloted with a small group (n=8) to ensure clarity, appropriate difficulty, and the absence of floor or ceiling effects [34]. The reading comprehension materials and the volume control interface are available at [35].

#### D2 Test of Attention

The d2 Test of Attention [36] will be used to assess selective and sustained attention under different auditory conditions. Participants scan rows of characters and mark specific target symbols (the letter “d” with two dashes) while ignoring similar distractors (eg, “p” with two dashes or “d” with one dash). This task is sensitive to attentional performance and processing speed, as it requires rapid visual discrimination and the suppression of irrelevant stimuli. This task was chosen over alternatives such as the Ruff 2 & 7 Test [37] due to its strong psychometric validity, widespread use, and accessibility.

### Auditory Stimuli and Volume Control

#### Overview

To define and validate the background audio conditions, several candidate voice-based audio samples were systematically evaluated to create 2 perceptually distinct auditory stimuli. Focused vocal stimuli are hypothesized to capture attention and disrupt performance, and diffused vocal stimuli are hypothesized to function as a nondistracting background.

For the focused vocal stimuli condition, a composite audio sample was created using Wikitongues [38] recordings in multiple languages unfamiliar to participants (eg, Quechua, Malagasy, and Basque). This approach maximizes prosodic and phonetic variability while minimizing semantic intelligibility, incorporating diverse phonemes, formant patterns, and speaking styles to increase perceived attention capture without introducing comprehensible linguistic content.

For the diffused vocal stimuli condition, ambient recordings of human chatter were collected from sources such as job fairs, cafés, and libraries. These were selected by the research team because such recordings were perceived as soothing. These recordings featured multiple overlapping voices with limited semantic intelligibility. After initial selection, multiple 10-second samples were extracted and evaluated informally by a group of listeners (n=6). One sample was consistently rated as the least distracting and was selected for use in the experiment.

Both audio samples were embedded in a custom HTML interface allowing participants to adjust playback volume within predefined limits (20‐65 dB).

#### Physiological and Pupillometric Measures

Physiological data will be collected using a combination of wearable sensors and environmental monitoring tools. Peripheral physiological signals including EDA, BVP, skin temperature, and motion will be recorded using the Empatica EmbracePlus (Empatica Inc.) wristband, worn on the nondominant wrist to minimize movement artifacts. The device will be adjusted before each session to ensure a secure fit without restricting blood flow, and electrode contact will be verified to prevent signal degradation.

Pupillometric data will be collected using the Pupil Core headset (Pupil Labs), with gaze information used to support calibration and data-quality assessment. Gaze calibration will be performed using a printed A3-sized calibration sheet containing a fixed grid of targets. Participants will be instructed to fixate on each target sequentially to establish accurate gaze mapping on the printed materials.

Ambient lighting will be monitored using a TopTes TS-710 Digital Lux Meter (Toptes), with adjustments made as needed to ensure consistent illumination across sessions. To verify background audio intensity, decibel levels from the speakers will be measured using a TopTes TS-501B Sound Level Meter in slow-response mode. Participants will be allowed to adjust the volume using a custom HTML interface featuring a constrained slider with a predefined range of 20‐65 dB. This range was selected to reflect ecologically valid indoor sound levels while avoiding discomfort or stress, while also preventing participants from nullifying the auditory manipulation. The selected volume levels will be recorded as subjective indicators of perceived comfort under each auditory condition.

Table 2 summarizes the schedule of measures and assessments by study phase.

**Table 2.** Schedule of measures and assessments by study phase.

Measure or assessment	Screening	Pre-experimental	Baseline	Block 1^a^	Block 2^a^	Block 3^a^	Postsession	Role in analysis
Eligibility screening	✓							Descriptive
Informed consent	✓							
Demographic questionnaire (6 items)		✓						Descriptive
µMCTQ^b^ (6 items)		✓						Descriptive, exploratory
PSQI^c^ (19 items)		✓						Exploratory moderator (H3)
WNSS-21^d^ (21 items)		✓						Exploratory moderator (H3)
ASRS-v1.1^e^ (18 items)		✓						Exploratory moderator (H3)
ARHQ-Brief^f^ (6 items)		✓						Descriptive, exploratory
BMIS^g^ (16 items)		✓						Exploratory moderator (H3)
Sensor setup and calibration		✓						
Silent signal-quality baseline			✓					Data-quality assessment
N-back task			✓					Exploratory moderator (H3)
Within-condition physiological baseline				✓	✓	✓		Baseline correction
Reading comprehension task				✓	✓	✓		Secondary behavioral outcome
d2 Test of Attention (CP score)^h^				✓	✓	✓		Primary confirmatory outcome (H1)
ESSQ-B^i^ (7 items)				✓	✓	✓		Manipulation check, descriptive
Perceived distraction and fatigue				✓	✓	✓		Manipulation check, descriptive
Selected playback volume (dB)				✓^j^	✓^j^	✓^j^		Descriptive
Pupillometry (continuous)		✓	✓	✓	✓	✓		Secondary physiological outcome (H2)
EDA^k^ and BVP^l^ (continuous)		✓	✓	✓	✓	✓		Secondary physiological outcome (H2)
Open-ended debriefing feedback							✓	Descriptive

a Blocks 1‐3 correspond to the focused vocal, diffused vocal, and silence conditions, with condition order counterbalanced across participants.

b µMCTQ: Ultra-Short Version of the Munich ChronoType Questionnaire.

c PSQI: Pittsburgh Sleep Quality Index.

d WNSS-21: Weinstein Noise Sensitivity Scale.

e ASRS-v1.1: Adult ADHD Self-Report Scale.

f ARHQ-Brief: Abbreviated Adult Reading History Questionnaire.

g BMIS: Brief Mood Introspection Scale.

h CP: Concentration Performance.

i ESSQ-B: Emotional Salience of Sounds Questionnaire B.

j Selected playback volume is recorded only for the focused and diffused vocal conditions, not silence.

k EDA: electrodermal activity.

l BVP: blood volume pulse.

### Data Analysis

The primary objective of the analysis will be to determine whether attentional performance differs between the focused and diffused vocal conditions. Secondary analyses will examine the effects of auditory condition on reading comprehension and physiological responses. Exploratory analyses will examine whether individual differences, including noise sensitivity, ADHD symptoms, sleep quality, mood, and working memory performance, may moderate these effects.

#### Outcomes and Analysis Overview

The primary outcome will be performance on the d2 Test of Attention, operationalized as the CP score. Reading comprehension accuracy will be treated as a secondary behavioral outcome.

For H2, physiological responses will be examined at 2 levels: physiological state during the within-condition baseline and task-related change relative to that baseline. The three prespecified secondary physiological outcomes will be mean pupil diameter, phasic EDA response rate, and RMSSD. Other physiological features, including additional heart rate variability (HRV) indices and blink rate, will be treated as exploratory.

Table 3 summarizes the outcome measures, their designations, analysis models, and multiplicity strategies.

**Table 3.** Outcome measures, designation, analysis model, and multiplicity strategy.

Outcome	Designation	Model	Multiplicity strategy
d2 CP^a^ score	Primary confirmatory outcome (H1)	Linear mixed-effects model with auditory condition and block position as fixed effects and participant as a random intercept	Focused vs diffused: primary contrast, α=.05 without adjustment; focused vs silence and diffused vs silence: Holm-adjusted
Reading comprehension accuracy (item level)	Secondary behavioral outcome	Generalized linear mixed-effects model with binomial distribution and logit link; crossed random intercepts for participant and item	No multiplicity adjustment
Mean pupil diameter	Secondary physiological outcome (H2)	Linear mixed-effects model	Holm adjustment across the 3 prespecified H2 physiological outcomes
Phasic EDA^b^ response rate	Secondary physiological outcome (H2)	Linear mixed-effects model	Holm adjustment across the 3 prespecified H2 physiological outcomes
RMSSD^c^ derived from BVP^d^	Secondary physiological outcome (H2)	Linear mixed-effects model	Holm adjustment across the 3 prespecified H2 physiological outcomes
Task completion time	Exploratory	Linear mixed-effects model on log scale	No multiplicity adjustment
Additional pupillometry, EDA, and HRV^e^ features	Exploratory	Linear mixed-effects models as appropriate to outcome distribution	No multiplicity adjustment
Condition × individual-difference interactions (WNSS-21^f^, ASRS-v1^g^.1, PSQI^h^, N-back, BMIS^i^)	Exploratory (H3)	Separate mixed-effects models including condition × moderator interaction	No multiplicity adjustment
ESSQ-B^j^ ratings	Manipulation check	Descriptive statistics and within-subject comparisons	No multiplicity adjustment
Perceived distraction and fatigue	Manipulation check	Descriptive statistics and within-subject comparisons	No multiplicity adjustment

a CP: Concentration Performance.

b EDA: electrodermal activity.

c RMSSD: root-mean-square of successive differences.

d BVP: blood volume pulse.

e HRV: heart rate variability.

f WNSS-21: Weinstein Noise Sensitivity Scale.

g ASRS-v1.1: Adult ADHD Self-Report Scale.

h PSQI: Pittsburgh Sleep Quality Index.

i BMIS: Brief Mood Introspection Scale.

j ESSQ-B: Emotional Salience of Sounds Questionnaire B.

#### Missing Data Handling

Missing data will be evaluated for their extent and patterns. Questionnaire items will be administered electronically with completion checks to minimize item-level missingness. If a questionnaire nevertheless contains missing responses, it will be scored according to the published instrument-specific scoring instructions. Where a valid score cannot be calculated, the questionnaire score will be treated as missing for analyses involving that measure.

Physiological data quality will be assessed separately for pupillometry, EDA, and BVP using prespecified modality-specific quality criteria.

For pupillometry, blink-related and invalid samples will first be identified and removed. Samples immediately surrounding detected gaps will also be excluded to reduce partial-occlusion artifacts. Missing intervals of ≤500 ms will be reconstructed using linear interpolation between valid samples immediately preceding and following the gap, and longer gaps will not be interpolated [39-41]. Analysis segments containing <80% valid or interpolatable pupil samples will be excluded. The 80% threshold is adopted as a conservative prespecified quality criterion, as there is no universally established threshold for the permissible proportion of missing pupil data [42].

EDA data will be screened for signal loss and nonphysiological artifacts, including abrupt changes associated with movement, nonpositive skin conductance values, and discontinuities inconsistent with physiological responses [43]. Artifactual intervals will be removed before decomposition into tonic and phasic components. Analysis segments containing <80% artifact-free EDA samples will be excluded. The 80% threshold is adopted as a conservative prespecified quality-control criterion to ensure that physiological features are derived predominantly from artifact-free data. Phasic EDA features will be calculated from the remaining artifact-free portions of each segment.

BVP recordings will be processed to identify pulse peaks and derive interbeat intervals (IBIs). Implausible intervals and intervals affected by motion or pulse-detection errors will be identified and removed or corrected according to the prespecified preprocessing procedure. RMSSD will be calculated only for segments containing at least 60 seconds of usable IBI data, consistent with recommendations for short-term assessment of vagally mediated heart rate variability when longer recordings are not feasible [44]. Segments that do not meet this criterion will be excluded from RMSSD analysis. Frequency-domain and nonlinear HRV features will also be derived from the cleaned IBI data based on their potential relevance demonstrated in previous physiological studies [45,46], but will be treated as exploratory outcomes.

#### Physiological Signal Preprocessing and Temporal Alignment

Physiological recordings will be temporally aligned with the experimental procedure using timestamped event markers recorded with the Empatica EmbracePlus wristband. Event tags will be created at predefined points, including the beginning and end of each baseline and task segment, and matched to the corresponding eye-tracking recordings during preprocessing. Markers recorded at the beginning and end of each session will also be used to assess and, where necessary, correct for clock offset and drift between devices.

Recordings will then be segmented into the 3 experimental blocks, each comprising a within-condition baseline followed by the reading comprehension and d2 tasks under the same auditory condition. Within each block, physiological and pupillometric signals will be further segmented into the baseline, reading encoding, reading retrieval, and d2 task periods.

Pupillometry, EDA, and BVP signals will undergo modality-specific preprocessing to reduce noise and artifacts. Pupil-diameter signals will be filtered using a Butterworth low-pass filter with a cutoff frequency of 4 Hz [41], with blink-related and invalid samples handled according to the procedures described previously. EDA signals will be screened for artifacts and decomposed into tonic and phasic components using continuous decomposition analysis [47]. BVP signals will be processed to identify pulse peaks and derive interbeat intervals for HRV analysis.

Features will be extracted separately for each segment and auditory condition. Mean pupil diameter will be the principal pupillometric measure, phasic response rate the principal EDA measure, and RMSSD the prespecified cardiac measure. Additional physiological features, including other time-domain, frequency-domain, and nonlinear measures, will be treated as exploratory.

#### Physiological Baseline Correction

Each experimental block will begin with a within-condition physiological baseline collected under the same auditory condition as the subsequent reading comprehension and d2 tasks. Physiological responses will be analyzed at 2 levels. First, the within-condition baseline periods will be compared across the focused, diffused, and silence conditions to characterize tonic physiological differences associated with auditory exposure. Second, task-related physiological responses will be baseline-corrected using the baseline collected at the beginning of the corresponding experimental block, which will allow changes during the reading and d2 tasks to be evaluated relative to the participant’s physiological state under that auditory condition.

For pupillometry, baseline-corrected pupil diameter will be calculated by subtracting the mean pupil diameter during the within-condition baseline from the mean pupil diameter during the corresponding task segment. For EDA, tonic and phasic activity will be quantified separately, with task-related phasic responses being evaluated relative to the corresponding within-condition baseline. For cardiac measures, RMSSD and other prespecified heart rate variability indices will be calculated separately for the baseline and task segments, and task-related changes will be expressed relative to the within-condition baseline where appropriate.

The session-level silent recording obtained during sensor setup and calibration will be used for signal-quality assessment and descriptive reference purposes rather than for baseline correction of the primary physiological analyses.

#### Confirmatory Analysis for Primary Outcome

The primary analysis will use a linear mixed-effects model with d2 CP score as the dependent variable, auditory condition as a three-level within-participant fixed effect, block position as a covariate, and participant as a random intercept: CP∼condition + block position+(1∣participant).

Auditory condition will be entered as a 3-level categorical variable representing focused vocal stimuli, diffused vocal stimuli, and silence. Block position will be included as a numeric covariate to account for potential order-related effects. The model will be fitted in Python using the statsmodels package, and model estimates will be reported with 95% CIs.

The prespecified primary contrast will compare the focused and diffused vocal conditions. This contrast directly tests H1 and will be evaluated at a two-sided significance level of α=.05. Two additional contrasts, focused vs silence and diffused vs silence, will be conducted as secondary comparisons to aid interpretation of the condition effects. These 2 secondary contrasts will be adjusted for multiple testing using the Holm procedure to control the family-wise error rate [48,49]. Estimated marginal means, contrast estimates, standardized effect sizes, and 95% CIs will be reported.

#### Secondary Behavioral Analysis

Reading comprehension accuracy will be analyzed as a secondary behavioral outcome at the item level using a generalized linear mixed-effects model with a binomial distribution and logit link. The model will include auditory condition and block position as fixed effects and crossed random intercepts for participant and item. The same three condition contrasts will be estimated, with focused vs diffused providing the principal comparison and comparisons with silence providing additional context.

#### Secondary Physiological Analysis

Physiological responses will be analyzed separately for within-condition baseline periods and task periods. First, physiological measures obtained during the baseline period of each block will be compared across the focused, diffused, and silence conditions using mixed-effects models with auditory condition and block position as fixed effects and participant as a random intercept. These analyses will examine whether physiological state differs across auditory conditions before task performance.

Second, physiological responses during the reading comprehension and d2 tasks will be evaluated relative to the within-condition baseline of the corresponding block. Baseline-corrected task responses will be analyzed using mixed-effects models with auditory condition, task segment, and block position as fixed effects and participant as a random intercept. Where relevant, the condition × task segment interaction will be examined to determine whether condition-related physiological differences vary between the reading and d2 tasks.

Associations between physiological responses and behavioral performance will be examined as exploratory analyses and reported using effect estimates and 95% CIs.

#### Exploratory Moderation and Other Exploratory Analyses

Individual-difference measures, including noise sensitivity, ADHD symptoms, sleep quality, mood, and N-back performance, will be examined as standardized continuous moderators by adding condition × moderator interaction terms to the mixed-effects models.

Because the study is powered for the primary focused-vs-diffused contrast rather than interaction effects, the moderation analyses will be considered exploratory and hypothesis-generating. Interaction estimates and 95% CIs will be reported to identify potential moderators for future investigation.

Exploratory analyses, including additional physiological features, moderation analyses, physiological-behavioral associations, and machine-learning analyses, will also be conducted to generate hypotheses for future research. These analyses will emphasize effect estimates and 95% CIs rather than dichotomous interpretation based on statistical significance.

#### Multiple Comparisons

The focused-vs-diffused contrast for the primary d2 CP outcome constitutes the single prespecified confirmatory test and will therefore be evaluated at α=.05 without adjustment for multiplicity. The 2 secondary comparisons of focused vs silence and diffused vs silence will be adjusted using the Holm procedure. For the 3 prespecified secondary physiological outcomes (mean pupil diameter, phasic EDA response rate, and RMSSD), *p*-values will also be adjusted using the Holm procedure to control the family-wise error rate.

#### Model Diagnostics and Sensitivity Analyses

Descriptive statistics and distributions will be reported for all outcomes. Assumptions of the linear mixed-effects models will be assessed through inspection of residual and quantile-quantile plots [50]. Linear mixed-effects models are generally robust to moderate violations of distributional assumptions, although substantial violations may affect the precision and validity of inference [51]. Where substantial violations are identified, sensitivity analyses using appropriate alternative specifications or robust methods will be conducted to assess whether the direction and magnitude of the primary focused-vs-diffused contrast are substantially affected [52,53]. The prespecified primary analysis will remain the principal analysis, and any sensitivity analyses and their rationale will be reported separately.

### Grouping of Harms and Reporting of Adverse Events

The study poses minimal risk. The principal foreseeable burdens were fatigue and possible discomfort from the auditory stimuli, both of which were mitigated by design. Playback volume will not exceed 65 dB, participants will set the volume themselves within the permitted range, and rest intervals will separate the experimental blocks. Participants will be reminded at the start of the session that they may pause or discontinue participation at any time, and the researcher (C. D. K.) will monitor for signs of fatigue or discomfort throughout the session. Any unexpected or serious adverse event related to study participation will be documented and reported to the Ethics Review Board in accordance with institutional procedures.

### Ancillary and Posttrial Care

Ancillary and posttrial care requirements are expected to be limited given the minimal-risk nature of the study. Skin irritation or minor discomfort associated with wearing the wristband or eye-tracking headset will be addressed immediately by the study staff, and participants will be referred to the university nursing office if further attention is needed. Any study-related harm will be managed in accordance with the institutional policies of Kyoto University of Advanced Science. No compensation will be provided for health events unrelated to study participation.

### Protocol Amendments

Any substantive amendment to the protocol, including changes to eligibility criteria, study procedures, outcomes, or the analysis plan, will be submitted to the Ethics Review Board of Kyoto University of Advanced Science (KUAS) for approval before implementation. Approved amendments will be documented with the date, nature, and rationale for the change and communicated to study staff before implementation. Where an amendment affects information provided to participants or study procedures requiring consent, the participant information sheet and consent form will be revised and submitted for approval accordingly. All substantive amendments will be reported in the Registered Report Stage 2 manuscript. Any deviation from the prespecified confirmatory analysis plan will be clearly identified and justified, and the affected analyses will be reported as exploratory. Administrative changes that do not affect study conduct, participant safety, or the analysis plan will be documented internally and handled according to institutional requirements.

## Results

### Principal Findings

Ethics approval for the study was granted by the Ethics Review Board of KUAS (approval number 26E01) on June 22, 2026. The study received no external funding. The protocol was finalized following the formative pilot described in the Introduction, which was conducted with 9 participants between January and July 2025. No participants have been recruited for the full study described in this protocol. Recruitment is scheduled to begin in September 2027, with data collection projected to conclude in September 2029. Data analysis is expected to be completed by September 2030. Dissemination of the results is anticipated by September 2031.

### Dissemination Policy

Results of the full study will be reported in a subsequent peer-reviewed publication. Authorship will follow the International Committee of Medical Journal Editors (ICMJE) criteria, and no professional writers will be used. Deidentified participant-level behavioral and questionnaire data and derived physiological features will be deposited in PhysioNet (MIT Laboratory for Computational Physiology) [54], subject to the study’s ethics approval and data-sharing requirements. Analysis code will be made available through the project GitHub (GitHub, Inc) repository to support reproducibility. Findings will also be presented at relevant conferences in human-computer interaction and psychophysiology. Participants who wish to receive the study results will be provided with a plain-language summary by email.

## Discussion

### Principal Results

This paper presents an experimental protocol designed to investigate whether background human speech with distinct paralinguistic characteristics, operationalized as focused vs diffused vocal stimuli, influences attentional performance. The protocol combines cognitive tasks, psychometric assessments, and physiological measurements in a counterbalanced within-subject design, which allows behavioral and physiological responses to be compared across auditory conditions while accounting for individual differences. The primary contribution of the protocol is the controlled manipulation of paralinguistic characteristics while minimizing semantic accessibility, providing an approach to investigating the role of nonsemantic vocal features in auditory distraction. Concurrent physiological measurements will complement the behavioral outcomes by examining physiological responses associated with auditory condition and attentional performance.

### Comparison With Prior Work

Previous research on noise and cognitive performance has primarily examined music, stationary nonspeech noise such as white or pink noise, or semantically intelligible speech, typically using behavioral outcomes such as accuracy and response time [1,2,5-9]. Theoretical frameworks offer different explanations for the effects of background sound. The MBA model proposes that moderate levels of noise may enhance or impair attention depending partly on baseline arousal and individual characteristics [13], whereas interference accounts emphasize disruption arising from changing-state acoustic structure and attentional capture [2,6,8]. However, comparatively little research has isolated the paralinguistic properties of human speech, such as prosodic and rhythmic variation, from its semantic content.

Research on background speech and cognitive performance has also relied primarily on behavioral measures, which provide limited information about physiological responses accompanying attentional performance. The present protocol combines established psychophysiological measures, including pupillometry, EDA, and BVP, with behavioral outcomes [14-16] to examine whether paralinguistic characteristics of semantically inaccessible background speech are associated with differences in attentional performance and physiological responses. The assessment of individual differences will further allow exploratory investigation of factors that may contribute to variation in susceptibility to auditory distraction.

### Implications for Research and Practice

The study may have implications for 3 related areas, although these implications will depend on the observed findings and should therefore be considered prospective. First, for research on auditory distraction, experimentally manipulating paralinguistic configuration while minimizing semantic accessibility would provide a means of examining whether nonsemantic properties of background vocal stimuli contribute to attentional disruption [2,6,8]. A difference in attentional performance between the focused and diffused conditions would provide initial evidence that attentional performance differs between the paralinguistic configurations examined and would motivate further investigation of the acoustic and attentional mechanisms underlying this difference. Conversely, little or no difference between the conditions would suggest that the specific paralinguistic manipulation examined here has limited influence on attentional performance under the tested conditions. In either case, the findings may help refine hypotheses concerning how the structural properties of background speech interact with attention.

Second, the findings may have implications for research on the design of occupational and educational environments. Background speech is common in shared offices, classrooms, libraries, and study spaces, and responses to such environments may vary according to individual characteristics, including noise sensitivity and ADHD symptomatology [4,5,7]. Evidence that particular paralinguistic configurations are associated with greater attentional disruption could inform future research on acoustic environments, including acoustic treatment, quiet-space provision, and masking or ambient sound [9,55]. The exploratory moderation analyses may highlight individual characteristics for further investigation. The present study is not designed to establish individualized accommodation recommendations but may help identify questions for subsequent, adequately powered research.

Third, the combination of behavioral and wearable physiological measurements may inform future research on adaptive sound environments and digital interventions. If physiological changes are associated with auditory condition or attentional performance, they could provide candidate markers for subsequent investigation in adaptive soundscape systems, focus-support applications, or context-aware interventions [15,16,55]. The present protocol provides a controlled setting for evaluating these associations. Determining whether such signals can support reliable individual-level prediction or real-time adaptation would require further validation in larger samples and real-world settings.

### Strengths of the Protocol

A key strength of the protocol is its controlled manipulation of paralinguistic characteristics while minimizing semantic accessibility. Comparing focused and diffused vocal stimuli within the same participants will provide a means of examining whether differences in nonsemantic vocal characteristics are associated with attentional performance while reducing between-participant variability. The inclusion of a silence condition will additionally provide a reference against which the effects of both vocal conditions can be evaluated.

The protocol is also designed to support replication and adaptation. The central auditory manipulation can be applied to different participant populations and cognitive tasks, while psychometric measures can be adapted to characteristics relevant to the population under investigation. Making the reading materials, auditory stimulus samples, and volume-control interface openly available will further support replication and extension of the study.

The use of behavioral and physiological measures will provide complementary information about responses to the auditory conditions. Behavioral outcomes will assess whether performance differs across conditions, whereas pupillometry, EDA, and BVP-derived measures will allow secondary and exploratory examination of accompanying physiological responses. The protocol therefore will provide a basis for investigating whether behavioral differences, if observed, are accompanied by measurable physiological changes.

### Limitations

Several limitations should be acknowledged. First, participants will select the playback volume within a predefined range of 20‐65 dB. Although this approach balances experimental control with participant comfort, it may introduce variability in perceived sound intensity across participants. Playback level will therefore be recorded to characterize between-participant variation in stimulus intensity and may be examined in exploratory sensitivity analyses where appropriate. Second, some procedures will require manual execution, including timing of the d2 Test of Attention and scoring of responses. Standardized instructions and scoring procedures will be used to minimize experimenter-related variability, but greater automation could improve precision in future implementations. Third, the reading comprehension task was developed specifically for this study and has undergone only formative pilot testing. The pilot was used to assess task feasibility and identify potential floor or ceiling effects rather than to establish psychometric validity or reliability. Its measurement properties will therefore require further evaluation using data from the full study. Finally, the study is powered for the prespecified focused-vs-diffused contrast on the primary d2 CP outcome. The sample size was not determined specifically for the secondary physiological outcomes or exploratory moderator interactions, and the study may therefore have limited power to detect effects in these analyses. Findings from these analyses will require confirmation in appropriately powered future studies.

### Conclusions

This protocol provides a controlled approach to investigating whether paralinguistic characteristics of semantically inaccessible background speech influence attentional performance. By comparing focused and diffused vocal stimuli within a counterbalanced within-subject design, the study will test their effects on the primary behavioral outcome while examining secondary behavioral and physiological responses and exploring potential individual differences. The findings may contribute to understanding auditory distraction and inform future research on the design of occupational, educational, and adaptive sound environments.

## Supplementary material

10.2196/93525 Checklist 1 SPIRIT 2025 checklist, applied where relevant to the present nonrandomized within-subject experimental protocol.

## Abbreviations

ADHD: attention-deficit hyperactivity disorder
ARHQ: Adult Reading History Questionnaire
ASRS: Adult ADHD Self-Report Scale
BMIS: Brief Mood Introspection Scale
BVP: blood volume pulse
CP: concentration performance
EDA: electrodermal activity
ESSQ-B: Emotional Salience of Sounds Questionnaire B
HRV: heart rate variability
IBI: interbeat intervals
ICMJE: International Committee of Medical Journal Editors
KUAS: Kyoto University of Advanced Science
MBA: moderate brain arousal
PSQI: Pittsburgh Sleep Quality Index
RMSSD: root-mean-square of successive differences
SPIRIT: Standard Protocol Items: Recommendations for Interventional Trials
WNSS-21: Weinstein Noise Sensitivity Scale
µMCTQ: Ultra-Short Version of the Munich ChronoType Questionnaire
